# The dual role of mesenchymal stem cells in apoptosis regulation

**DOI:** 10.1038/s41419-024-06620-x

**Published:** 2024-04-06

**Authors:** Zhuo Chen, Xuewei Xia, Mengwei Yao, Yi Yang, Xiang Ao, Zhaoqi Zhang, Li Guo, Xiang Xu

**Affiliations:** 1 Department of Stem Cell & Regenerative Medicine, State Key Laboratory of Trauma and Chemical Poisoning, Daping Hospital, Army Medical University, Chongqing, 400042 China; 2 Department of General Surgery, The 906th Hospital of PLA, Ningbo, 315040 Zhejiang China; 3 Department of Biochemistry and Molecular Biology, College of Basic Medical Sciences, Army Medical University, Chongqing, 400042 China; 4 Department of Rheumatology and Immunology, Daping Hospital, Army Medical University, Chongqing, 400042 China; 5 Department of orthopedics, The 953th Hospital of PLA, Shigatse Branch of Xinqiao Hospital, Army Medical University, Shigatse, 857000 China; 6 Department of Neurosurgery, The 906th Hospital of PLA, Ningbo, 315040 Zhejiang China; 7 Endocrinology Department, First Affiliated Hospital, Army Medical University, Chongqing, 400038 China; 8 Yunnan Key Laboratory of Stem Cell and Regenerative Medicine, Science and Technology Achievement Incubation Center, Kunming Medical University, Kunming, 650500 China

**Keywords:** Mesenchymal stem cells, Apoptosis

## Abstract

Mesenchymal stem cells (MSCs) are widely distributed pluripotent stem cells with powerful immunomodulatory capacity. MSCs transplantation therapy (MSCT) is widely used in the fields of tissue regeneration and repair, and treatment of inflammatory diseases. Apoptosis is an important way for tissues to maintain cell renewal, but it also plays an important role in various diseases. And many studies have shown that MSCs improves the diseases by regulating cell apoptosis. The regulation of MSCs on apoptosis is double-sided. On the one hand, MSCs significantly inhibit the apoptosis of diseased cells. On the other hand, MSCs also promote the apoptosis of tumor cells and excessive immune cells. Furthermore, MSCs regulate apoptosis through multiple molecules and pathways, including three classical apoptotic signaling pathways and other pathways. In this review, we summarize the current evidence on the regulation of apoptosis by MSCs.

## Facts

- MSCs protect tissue cells from apoptosis and improve diseases through apoptosis regulation pathways.
- MSCs promote specific cell apoptosis to combat autoimmune diseases and tumors.
- The apoptosis of MSCs themselves or surrounding tissue cells also helps enhance the therapeutic effect.

## Open Questions

- What are the most key molecules for the antiapoptotic effect of MSCs, and how to stabilize and enhance the antiapoptotic effect of MSCs?
- What are the effects of MSCs on other forms of programmed cell death?
- How to explore the intrinsic mechanisms and potential clinical application strategies of apoptosis in MSCs?
- How to enhance the therapeutic effects of MSCs through apoptotic products?

## Introduction

Mesenchymal stem cells (MSCs) transplantation therapy (MSCT) has been widely recognized as an effective clinical strategy for a variety of diseases [1]. And the therapeutic effect of MSCs mainly depends on several factors and molecules, including immunomodulatory molecules, chemokines, growth factors, and non-coding RNAs (ncRNAs) [2–4]. The factors and molecules affect the proliferation, apoptosis, immune homeostasis and metabolic balance of disease-related cells. Through the mediators, MSCs reduce disease damage and improve regeneration potential by inhibiting abnormal apoptosis and promoting tissue cell proliferation. In addition, MSCs have a strong immunoregulatory capacity, which effectively regulate the immune homeostasis of disease tissues and protect normal tissue cells from damage [1, 5]. However, there remains a lack of understanding of the above processes, which hampers the further clinical application of MSCs.

Apoptosis is a complex programmed cellular death process that involves many molecules and pathways [6]. Physiological apoptosis maintains homeostasis by removing senescent cells and abnormal cells, but pathological apoptosis is an important factor in the occurrence and development of diseases [7]. In ischemic-reperfusion injuries, hemorrhagic diseases, and neurodegenerative diseases, excessive apoptosis of cells is a key factor in the development of the diseases [8–10]. In addition, resistance of tumor cells to apoptosis is an important contributor to tumor proliferation and invasion [11, 12]. Therefore, correcting the abnormal apoptosis process of cells is an important method to alleviate the diseases.

**Fig. 1:**
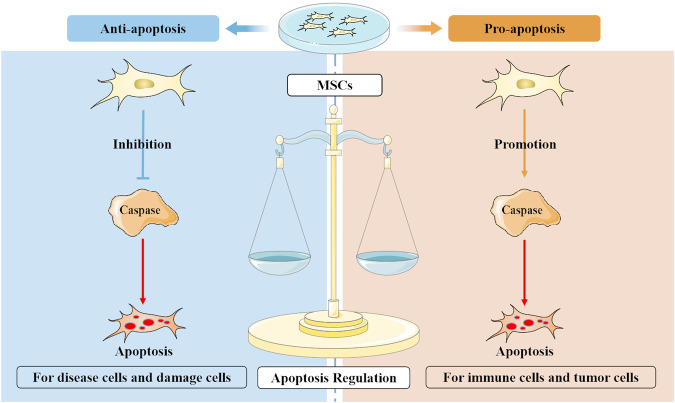
Mesenchymal stem cells (MSCs) exhibit varying apoptotic regulatory effects for different cell. The influence of MSCs on apoptosis varies depending on the target cells. MSCs typically demonstrate significant antiapoptotic effects on cells that have been damaged by disease or trauma. However, MSCs can also promote the apoptosis of immune cells and tumor cells. These findings highlight the crucial role of MSCs in the intricate regulatory network of apoptosis.

MSCs regulate the apoptosis of the cells through multiple molecules and pathways (Fig. 1). Many studies have found that MSCT significantly reduce the apoptosis of diseases-related tissue cells (Table 1), which is achieved by the activation of a variety of signaling pathways [13–15]. More interestingly, MSCs also alleviate diseases by promoting apoptosis in some cells, such as autoimmune diseases and tumors [16–18]. These evidences suggest that MSCs exert therapeutic effects by regulating the apoptotic process of various cells. In this review, we summarize the current understanding of the role of MSCs in the regulation of apoptosis, and provide a new perspective on the relationship between MSCs and apoptosis.

**Table 1:** Mechanism of MSCs resistance to apoptosis.

Pathways	Molecules	Mechanisms	Refs
**Mitochondrial pathway**	IL-6	Via the activation of the JAK-STAT3-Ref-1 and JAK-Stat3-Bcl-2/Bax-Caspase-3 pathways; Upregulate the mRNA expression of Cyclin D and Bcl-xl	[28, 37]
	PGE2	Via ERK1/2 and GSK3beta phosphorylation to increase Bcl-2 and decrease Bax expression	[29]
	TGF-β	Through TGF-β/Bax singling pathway	[30]
	miR-29a-3p	Regulate Bcl-2 and Bcl-xl genes	[31]
	miR-125b-5p	Repress the protein expression of p53, leading to the modulation of Bcl-2 and Bax to inhibit apoptosis	[32]
	miR-93	Through targeting the HDAC4/Bcl-2 axis	[33]
	miR-150-5p	Via targeting Bax	[34]
	lncRNA-UCA1	Target miR-873 via sponging, reducing the latter’s suppressive effects on its target XIAP, and this translated into AMPK phosphorylation and increased level of the antiapoptotic protein Bcl-2	[35]
		Enhance translocation of Bcl-2 to the nucleus	[36]
	CXCL12	Reduce caspase-3 activation and modulate the expression of the antiapoptotic protein Bcl-xl	[38]
		Upregulate p-AKT and p-Bad by PI3K-AKT-Bad pathway	[39]
**Death receptor pathway**		Suppress the protein expression levels of macrophage-related factors inducible nitric oxide synthase and TNF-α	[40]
	miR-21-5p	Modulate Fas-L expression	[41]
	miR-17	Regulate BRD4-mediated EZH2/TRAIL axis to essentially inhibit LPS-induced macrophages inflammation and apoptosis	[42]
		Via TLR3-regualted MAPK and NF-κB signaling pathway	[43]
**Endoplasmic reticulum pathway**	HGF	Via a microenvironment-dependent paracrine HGF/c-Met signaling mechanism to suppress ERS and its downstream pro-inflammatory and pro-apoptotic consequences	[44]
	TNF-inducible gene 6 protein	Suppress ERS-induced apoptosis and NF-κB activity	[45]
	miR-21	By alleviating ERS and inhibiting p38 MAPK	[46]
		By improving Myc expression through both stromal cell-derived factor 1 signal and contact effect	[47]
**Upstream regulatory pathways**	miR-29b-3p	Activate the PI3K/AKT pathway by carrying miR-29b-3p into neurons and silencing PTEN, thus reducing neuronal apoptosis	[49]
	miR-223	Inhibit the apoptosis of neurons in vitro by targeting PTEN, thus activating the PI3K/Akt pathway	[50]
	miR-144	Inhibit cell apoptotic injury in hypoxic conditions by delivering miR-144 to cells, where it targets the PTEN/AKT pathway	[51]
	miR-486-5p	By suppressing PTEN expression, activating the PI3K/AKT signaling pathway, and subsequently inhibiting the apoptosis of injured cardiomyocytes	[52]
	lncRNA-KLF3-AS1	Inhibit autophagy and apoptosis of IL-1beta-treated chondrocyte through PI3K/Akt/mTOR signaling pathway	[53]
	miR-132-3p	Downregulate the target protein RASA1, while upregulate the expression of Ras and the downstream PI3K phosphorylation	[54]
		Involve NRG-1/HER2, MAPK, PI3K/AKT, p-JNK/JNK, and p-STAT/STAT signaling pathways	[162]
		Involve G-CSF/PI3K/AKT pathway	[163]
		By inhibiting apoptosis of skin cells and promoting their proliferation through activating PI3K/AKT signaling pathway	[164]
	miR-369-3p	Downregulate the expression of YAF2, inhibit the stability of PDCD5/p53, and reduce the apoptosis of ovarian granulosa cells	[55]
	miR-644-5p	Inhibit the apoptosis of ovarian granulosa cell by targeting p53	[56]
	miR-125b-5p	Suppress the expression of the pro-apoptotic genes p53 and BAK1 in cardiomyocytes	[57]
	miR-455-3p	Target the MEKK1-MKK4-JNK signaling pathway	[58]
	miR-19a	Target SOX6, activate AKT, and inhibit JNK3/caspase-3 activation	[59]
		By inhibiting p38/MAPK pathway	[60–62]
**Others**		By transferring mitochondria	[63, 64]
		Increase the mitochondrial membrane potential and alleviate compression-induced mitochondrial damage to alleviate compression-mediated nucleus pulposus cell apoptosis	[65]
		Retard mitochondria damage and cell apoptosis by an AMPK-PGC1-alpha axis	[66]
		Promote mitophagy and inhibit apoptosis and pyroptosis of renal tubular epithelial cells in kidney tissues by upregulating SIRT1/Parkin	[67]
		By reduce mitochondrial reactive oxygen species overproduction, decrease the accumulation of mitochondrial fragmentation, restore ATP generation and upregulate mitophagy	[68]
	miR-486	Reduce Smad1 expression by target regulating Smad1 whose reduction could inhibit mTOR activation, leading to the increase of autophagy and the reduction of podocyte apoptosis	[69]
		Through regulating Notch2/mTOR/autophagy signaling	[70]
	miR-217	Target EZH2, and EZH2 bound to the FOXO3 promoter and consequently downregulate its expression, which restrain NPC apoptosis and ECM degradation by stimulating cell autophagy	[71]
	ALKBH5	ALKBH5-mediated FIP200 mRNA demethylation in enhancing autophagy and reducing apoptosis	[72]
		Reduce pyroptosis in the injured liver and promote the expression of those factors related to liver regeneration, while they can inhibit the NF-κB pathway and activate the wnt/beta-catenin pathway	[73]
		Increase FOXO3a expression to enhance mitophagy, therefore protecting microglia from I/R-induced pyroptosis and alleviating subsequent neuronal injury	[74]
	miR-539-5p	Suppresses pyroptosis through NLRP3/caspase-1 signal	[75]
	circ-HIPK3	By regulate miR-421, resulting in increased expression of FOXO3a, leading to inhibition of pyroptosis and release of IL-1beta and IL-18	[76]
	miR-223-3p	Restrict cardiac inflammation, pyroptosis, and dysfunction by disrupting FOXO3/NLRP3 axis	[77]
	miR-26a-5p	Degrade METTL14 and thus decrease NLRP3	[78]
	circ-003564	Attenuate inflammasome-related pyroptosis via delivering circ-003564	[79]

*MSCs* mesenchymal stem cells, *IL* Lnterleukin, *JAK* Janus kinase, *STAT* signal transducer and activator of transcription, *Ref-1* Redoxfactor-1, *Bcl-2* B-cell lymphoma-2, *PGE2* prostaglandin E2, *ERK* extracellular regulated protein kinases, *GSK3β* glycogen synthase kinase 3, *TGF-β* transforming growth factor-beta, *HDAC* histone deacetylase, *miR* micro RNA, *XIAP* X-linked inhibitor of apoptosis protein, *AMPK* Adenosine 5′-monophosphate-activated protein kinase, *CXCL12* C-X-C motif chemokine 12, *PI3K* phosphatidylinositol 3-kinase, *TNF-α* Tumor necrosis factor-alpha, *Fas-L* Fas ligand, *BRD4* bromodomain-containing protein 4, *EZH2* enhancer of zeste homolog 2, *TRAIL* TNF-related apoptosis inducing ligand, *LPS* lipopolysaccharide, *TLR3* Toll-like receptor 3, *MAPK* mitogen-activated protein kinase, *HGF* hepatocyte growth factor, *ERS* endoplasmic reticulum stress, *PTEN* phosphatase and tensin homolog, *RASA1* Ras GTPase activating protein 1, *NRG-1* neuregulin-1, *HER2* human epidermal growth factor receptor-2, *G-CSF* granulocyte colony-stimulating factor, *YAF2* YY1 associated factor 2, *PDCD5* programmed cell death 5, *BAK1* Bcl-2 antagonist/killer 1, *MEKK1* MAPK kinase 1, *MKK4* MAPK kinase 4, *JNK* c-Jun N-terminal kinase, *SOX6* SRY-box transcription factor 6, *PGC1* peroxisome proliferator-activated receptor gamma coactivator 1, *SIRT1* Sirtuin 1, *EZH2* enhancer of zeste homolog 2, *FOXO3* forkhead box O3, *NPC* nucleus pulposus cells, *ECM*: extracellular matrix, *ALKBH5* ALKB homolog 5, *FIP200* FAK-family interacting protein of 200 kDa, *NLRP3* NOD-like receptor thermal protein domain associated protein 3, *METTL14* methyltransferase-like protein 14.

## MSCs protect cells from apoptosis

**Fig. 2:**
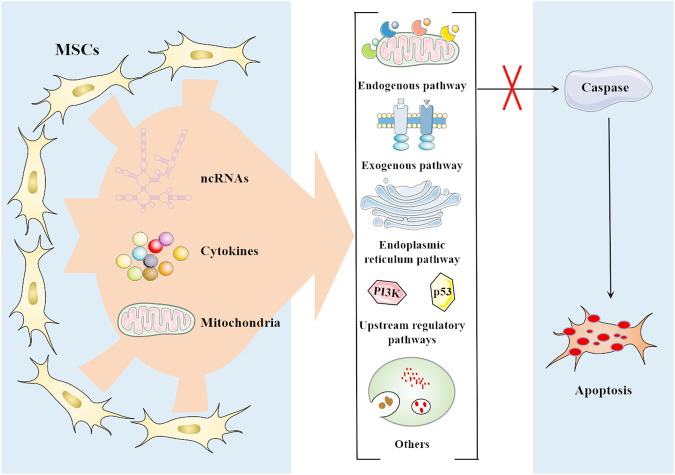
Mesenchymal stem cells (MSCs) inhibit apoptosis by targeting multiple pathways. MSCs of various origins influence the outcome of apoptosis by targeting multiple apoptosis regulatory pathways. This is mainly achieved through their secreted components, which include various ncRNAs and cytokines. These antiapoptotic mediators significantly inhibit caspase cascade in target cells through endogenous pathways, exogenous pathways, and endoplasmic reticulum stress pathways, thereby preventing apoptosis. Upstream signaling pathways of apoptosis regulation, such as the PI3K pathway and p53 pathway, are also among the targets of regulation. In addition, MSCs also exert antiapoptotic effects through the regulation of mitochondria and autophagy.

Inhibition of apoptosis is an important mechanism of MSCs to alleviate various diseases, including cardiovascular diseases, renal injury, neurodegenerative diseases, and premature ovarian failure [19–22]. In these diseases, the number of normal cells undergoing pathological apoptosis is closely related to the severity of the disease. Moreover, many studies have found that the apoptosis of these cells mainly through three pathways, including endogenous pathway, exogenous pathway, and endoplasmic reticulum pathway [23, 24]. And MSCs regulate the above three apoptotic pathways through various mechanisms to significantly reverse apoptotic events in various pathological states (Fig. 2).

### Endogenous pathway (mitochondrial pathway)

Endogenous pathway refers to apoptotic events induced by changes in mitochondrial membrane permeability, also known as mitochondrial pathway. The increased permeability of the mitochondrial membrane leads to the release of pro-apoptotic factors in the mitochondria into the cytoplasm, which activates the caspase cascade and initiates the apoptotic process [25, 26]. And b-cell lymphoma-2 (Bcl-2) family proteins are the main regulators of mitochondrial pathway, which regulate the permeability of mitochondrial membrane [27]. The Bcl-2 family includes antiapoptotic proteins Bcl-2 and Bcl-xl, pro-apoptotic proteins Bad, Bid, Bax, and Bim. And multiple factors derived from MSCs control the permeability of mitochondrial membrane and thus regulate apoptosis by regulating the differential expression of Bcl-2 family.

Bcl-2 and Bax are the most reported apoptotic indicator proteins. Many studies have found that multiple mediators derived from MSCs significantly increase antiapoptotic protein Bcl-2 and decrease pro-apoptotic protein Bax in disease cells, including lnterleukin-6 (IL-6), prostaglandin E2, transforming growth factor-beta, microRNA (miR)-29a-3p, and miR-125b-5p [28–32]. And miR-93, miR-150-5p, and long ncRNA-UCA1 from MSCs also promote the expression and recovery of Bcl-2 in target cells [33–35]. In addition, the conditioned medium of MSCs enhance translocation of Bcl-2 to the nucleus in mouse alveolar epithelial cells [36]. The findings suggest that MSCs have strong regulatory capabilities on both Bcl-2 and Bax. Moreover, MSCs also inhibit apoptosis by regulating the expression of Bcl-xl and Bad [37–39]. Therefore, the Bcl-2 family is an important center for the regulation of apoptosis by MSCs in the mitochondrial pathway.

### Exogenous pathway (Death receptor pathway)

Exogenous pathway refers to death receptor-mediated apoptotic events, also known as death receptor pathway. There are five major death receptors, including Fas, tumor necrosis factor receptor, death receptor (DR) 3, DR4, and DR5, and their corresponding ligands include Fas-L, tumor necrosis factor (TNF), DR3L, and TNF-related apoptosis inducing ligand (TRAIL). Specifically, the extracellular apoptotic signals activate the intracellular caspase cascade by activating different death receptors. And these death receptors and related signaling systems are also important targets for MSCs to exert antiapoptotic effects. For example, MSCT significantly reduce the TNF-α, inducible nitric oxide synthase, Fas-L, and other pro-apoptotic signals in the cell microenvironment, and finally alleviate the apoptosis of cells [40, 41]. Moreover, the miR-17 derived from MSCs-extracellular vesicles (EVs) regulates bromodomain-containing protein 4-mediated enhancer of zeste homolog 2 (EZH2)/TRAIL axis to essentially inhibit lipopolysaccharide (LPS)-induced inflammation and apoptosis of RAW264.7 cells [42]. Similarly, MSCs effectively inhibit alveolar macrophage apoptosis and reverse LPS-induced lung injury by reducing toll-like receptor (TLR) 3 mediated mitogen-activated protein kinase (MAPK) and NF-κB signaling [43]. These evidences suggest that MSCs inhibit death receptor-mediated apoptosis by reducing apoptotic signals and regulating death receptor apoptotic signaling pathway.

### Endoplasmic reticulum pathway

Endoplasmic reticulum stress (ERS) refers to an increase in misfolded proteins resulting from impaired endoplasmic reticulum function, and long-term ERS induce apoptosis [24]. Studies have found that hepatocyte growth factor (HGF) and TNF-inducible gene 6 protein secreted by MSCs significantly inhibit ERS and its subsequent pro-apoptotic and pro-inflammatory consequences [44, 45]. Moreover, miR-21 derived from MSCs-exosomes (MSCs-Exos) effectively inhibit hypoxia-induced apoptosis by alleviating ERS and inhibiting phosphorylation of p38 MAPK [46]. MSCs protect the islets after transplantation from ERS-induced apoptosis and improved the viability of the islets [47]. These findings reveal that MSCs can inhibit ERS and thus alleviate apoptosis.

### Upstream regulatory pathways

In addition to the three major apoptotic signaling pathways mentioned above, there are other pathways that control apoptosis by regulating the expression of survival and apoptosis-related genes. According to regulated genes, these pathways should be divided into two categories: antiapoptotic pathway and pro-apoptotic pathway. And these pathways are also important targets for MSCs to exert antiapoptotic effects.

### Antiapoptotic pathway

Phosphatidylinositol 3-kinase (PI3K)-AKT is an important pathway for cell survival, and its activation upregulate the expression of many antiapoptotic genes and proliferative genes [48]. Moreover, cancer suppressor gene PTEN dephosphorylates AKT and reduces its activation, which is a negative regulator of PI3K/AKT pathway. However, MSCs-derived miR-29b-3p, miR-223, miR-144, and miR-486-5p activate PI3K/AKT pathway by inhibiting PTEN, thus inhibiting apoptosis [49–52]. Similarly, long ncRNA KLF-AS1 and miR-132-3p derived from MSCs-Exos also activate the PI3K/AKT pathway and exert antiapoptotic effects [53, 54]. These findings show that MSCs protect cells from apoptosis by secreting various factors to activate the PI3K/AKT signaling pathway.

### Pro-apoptotic pathway

In the apoptotic signaling network, the p53 signaling pathway regulates the expression of pro-apoptotic genes, including Bax, Bak, Bad, and Apaf-1. However, miR-369-3p, miR-644-5p, and miR-125b-5p in MSCs-Exos exert antiapoptotic effects by inhibiting the activation of p53 [32, 55–57]. And interestingly, miR-455-3p and miR-19a exhibit antiapoptotic effects by inhibiting JNK and subsequent activation of p53 and caspase-3 [58, 59]. Additionally, MSCs significantly alleviate cisplatin-induced toxicity and improve islet viability by inhibiting p38/MAPK pathway [46, 60–62]. These findings indicate that MSCs synergistically exert an inhibitory effect on apoptosis by inhibiting the pro-apoptotic pathway while enhancing the antiapoptotic pathway.

### Others

MSCs also resist apoptosis in some interesting ways, including by regulating mitochondria and autophagy. Li, X et al. and Li, H et al. have found that MSCs protect airway smooth muscle cells and injured neurons from apoptosis by mitochondrial transfer [63, 64]. And MSCs also show the ability to regulate mitochondrial potential, reduce mitochondrial stress, and reduce mitochondrial damage to inhibit apoptosis [65, 66]. In addition, promoting mitochondrial protective autophagy is also an important way that MSCs resist apoptosis [67, 68]. Notably, the protective autophagy induced by MSCs is not only in the mitochondria but also in the whole cell. MSCs enhance autophagy flux by inhibiting mTOR signal activation, which promote autophagy and inhibit apoptosis [69, 70]. And forkhead box O3 and ALKBH5 are also targets of MSCs mediated protective autophagy to inhibit apoptosis [71, 72]. Therefore, MSCs fight apoptosis by regulating mitochondrial biological function and promoting protective autophagy.

Resistance to NOD-like receptor thermal protein domain associated protein 3 (NLRP3)-mediated pyroptosis is an interesting extension of the ability of MSCs to inhibit apoptosis. MSCT significantly decrease inflammasome-related pyroptosis markers including cleaved caspase-1, gasdermin D, NLRP3, IL-1beta, and IL-18 in diseases [73–75]. And MSCs-Exos also play a key role in this process. MSCs-Exos upregulate the expression of FOXO3a, which inhibit pyroptosis and the release of inflammatory cytokines [74, 76]. In MSCs-Exos, ciric-003564, miR-539-5p, ciric-HIPK3, miR-223-3p, and miR-26a-5p are all involved in the inhibition of NLRP3-mediated pyroptosis by MSCs [75–79]. These ncRNAs greatly enhance the remission effect of MSCs on various pyroptosis diseases, and suggest that MSCs have great potential to combat inflammatory apoptosis.

## MSCs improve diseases by inhibiting apoptosis

Inhibition of tissue cell apoptosis is a key mechanism in the therapeutic effect of MSCs, which enables MSCT to improve many diseases. These diseases involve the following tissues and organs, including heart, liver, lung, and nervous system. And Ischemia-reperfusion injury (IRI) is a common factor inducing abnormal apoptosis in these tissues and organs [80]. Interestingly, MSCs can significantly improve the tissue and organ dysfunction caused by these diseases through inhibiting abnormal apoptosis and inflammatory responses.

### Heart diseases

Myocardial infarction (MI) is a common heart disease in which myocardial cells undergo IRI, which often induces abnormal apoptosis of cardiomyocytes [81]. MSCs-derived medium and exosomes are considered as new biological drug for the treatment of MI. And various ncRNAs in the culture medium and exosomes have significant therapeutic effects by inhibiting cardiomyocyte apoptosis, including miR-150-3p, miR-144, miR-486-5p, miR-455-3p, miR-19a, miR-25-3p, miR-185, miR-221/222, and lncRNA-KLF3-AS1 [34, 51, 52, 58, 59, 82–85]. By secreting these ncRNAs, MSCT significantly inhibit myocardial cell apoptosis and fibrosis, reduce inflammation, and improve myocardial function. Notably, enhancing the cardioprotective effects of MSCs by genetic modification or gene editing also is a welcome approach [19]. In particular, various molecules with cardioprotective effects, include n-cadherin, lipocalin 2, c1q/tumor necrosis factor-related protein 3, follistatin-like 1, stromal-derived factor 1, v-erb-b2 avian erythroblastic leukemia viral oncogene homolog 4, and glucagon-like peptide-1 [86–92]. In addition, pretreatment of MSCs before transplantation in vitro also achieve similar enhancement effects by enhancing apoptosis resistance, such as hypoxia pretreatment, interferon (IFN)-γ pretreatment, atorvastatin pretreatment, sphingosine 1-phosphate pretreatment, and the combined pretreatment of HGF and insulin-like growth factor 1 [35, 93–96]. These findings suggest that enhancing the antiapoptotic ability of MSCs has a broad prospect in alleviating myocardial cell injury caused by MI.

### Liver diseases

Liver transplantation is an effective strategy for the treatment of various end-stage liver diseases; however, IRI of hepatocytes often occurs during this process [97]. Emerging evidences suggest that MSCs have a strong hepatoprotective effect, which helps to alleviate hepatic IRI, liver failure, and liver fibrosis [98–100]. The hepatoprotective effect is mainly reflected in inhibiting hepatocyte apoptosis, promoting hepatocyte proliferation, inhibiting liver inflammation, and oxidative stress. Various secretory mediators derived from MSCs significantly inhibit liver injury, improve the success rate of liver transplantation, promote liver regeneration, and improve liver function by playing a hepatoprotective effect [28, 101–103]. These mediators include IL-6, prostaglandin E2, ransforming growth factor-beta, and ncRNAs [28–30, 104]. In addition, heme oxygenase 1 modification and hypoxic preconditioning significantly enhance the hepatoprotective effect of MSCs [105, 106]. Therefore, MSCT has a significant alleviating effect on hepatocyte apoptosis caused by acute liver injury.

### Lung diseases

MSCT is also highly effective for various lung diseases through key mechanisms such as inhibition of apoptosis. Interestingly, the apoptotic protective effect of MSCs on alveolar epithelial cells has a broad range of disease applications, including acute respiratory distress syndrome or lung injury induced by IRI, smoke, influenza virus, sulfur mustard, and radiation [107–112]. In addition, MSCT also protect against pulmonary fibrosis induced by diabetes, silicosis, and bleomycin [113–115]. Moreover, the major protective mediators are mainly derived from the secretome of MSCs [36]. Therefore, MSCs have a wide range of effects to protect lung against various injury, including inhibiting alveolar epithelial cell apoptosis and fibrosis, inhibiting inflammation, reducing lung injury, and promoting the recovery of alveolar barrier function.

### Neurological diseases

Hypoxic-ischemic encephalopathy and spinal cord injury are important causes of neuronal apoptosis, which can also be mitigated by MSCs. In the in vivo rat middle cerebral artery occlusion model and in vitro neuronal oxygen and glucose deprivation experiments, MSCs significantly inhibit abnormal apoptosis of neuronal and microglial, and improve neurobehavioral deficits [33, 116]. And MSCs-derived exosomes or vesicles are the key mediators in this process [21]. In addition to secreting vesicles, an interesting mechanism by which MSCs alleviate spinal cord injury induced neuronal apoptosis is by mitochondrial transfer [117]. Mitochondria derived MSCs transfer to damaged neurons not only inhibit apoptosis but also promote axon regeneration, which improve motor recovery. Moreover, MSCs also alleviate Alzheimer disease by reducing inflammation, inhibiting apoptosis, and regulating autophagy [118].

### Other diseases

There are also diseases involving other organs or tissues that can be alleviated by MSCs through inhibiting apoptosis, including kidney injury, premature ovarian failure, pancreatitis, intervertebral disc degeneration, and osteoarthritis [67, 119–122]. Similar to the aforementioned diseases, MSCs significantly alleviate abnormal tissue cell apoptosis, reduce tissue inflammation and restore organ function in these diseases. In general, the antiapoptotic effect of MSCs has the following characteristics. The antiapoptotic ability of MSCs mainly alleviates two types of apoptosis-related diseases, instantaneous massive apoptosis due to acute injury and persistent apoptosis due to chronic injury. The remission of MSCs on these two apoptotic diseases mainly depends on blocking the apoptotic process and alleviating the inflammatory microenvironment. Moreover, the antiapoptotic mechanisms of MSCs are mainly mediated through their secretome, including ncRNAs, cytokines and mitochondria. MSCs secrete these mediators to inhibit apoptosis and reduce inflammation, which are the main therapeutic effects of MSCs on the disease. In addition, in vitro pretreatment or gene modification can enhance the effect of MSCT by enhancing the survival ability of MSCs or carrying protective components. These characteristics make MSCT have strong efficacy to be used in the treatment of many diseases.

## MSCs enhance apoptosis of target cells

### MSCs promote apoptosis of tumor cells

In addition to the aforementioned MSCs promote tumor growth by inhibiting tumor cell apoptosis, MSCs also are found to promote tumor cell apoptosis and inhibit tumor growth (Table 2). For example, miR-23b-5p derived from MSCs-Exos significantly reduce the proliferation and induce apoptosis of acute myeloid leukemia cells by reversing the TRIM14-activated PI3K/AKT pathway [123]. And miR-205 retards prostate cancer progression by inhibiting rhophilin Rho GTPase binding protein 2 [124]. Similarly, TNF-α-induced MSCs upregulate TRAIL expression and induce apoptosis in triple-negative breast cancer MDA-MB-231 (MDA) cells by secreting IFN-β [125]. And MSCs with highly expressed Fas-L significantly induce apoptosis in multiple myeloma cells [126]. Moreover, MSCs have also been shown to have significant pro-apoptotic effects on a variety of other tumor cells, including glioma U251 cells, pancreatic cancer cell, hepatocellular carcinoma cells, and lymphoma cells [127–130]. Therefore, MSCs have a strong pro-apoptotic effect on a variety of tumor cells through various pathways. These findings show the broad application prospect of MSCs in the field of tumor therapy; however, it also suggests that we need to further explore the relationship between MSCs and tumor cells.

**Table 2:** MSCs enhance apoptosis of target cells.

Molecules	Mechanisms	Effects	Refs
miR-23b-5p	By reversing the TRIM14-activated PI3K/AKT pathway	Reduce the proliferation and induce apoptosis of acute myeloid leukemia cells	[123]
miR-205	By inhibiting rhophilin Rho GTPase binding protein 2	Retard prostate cancer progression	[124]
IFN-β	By upregulating TRAIL expression	Induce apoptosis of MDA cells	[125]
Fas-L	By Fas/Fas-L pathway	Induce apoptosis of MM cells	[126]
	By downregulating the PI3K/AKT signaling pathway	Inhibit U251 cells proliferation and the EMT-like	[127]
	By altering cell cycle arrest and MMP7 signaling-triggered EMT	Inhibit PDAC cell proliferation, tumor growth and invasion	[128]
		Inhibit proliferation rate and increase the apoptosis rate	[129]
		Decrease cell viability and increase apoptosis	[130]
IDO		Inhibit T-cell proliferation by inducing apoptosis of activated T cells	[131]
PD-L1		Suppress the activation of CD4+ T cells, downregulate interleukin-2 secretion and induce irreversible hyporesponsiveness and cell death	[132]
Fas-L		Fas-regulated monocyte chemotactic protein 1 recruit T cells for Fas-L-mediated apoptosis	[18, 133]
	Through inhibiting TGF-β1 and Smad3 expression and increasing Smad7 protein expression	Promote hepatic stellate cells apoptosis	[134, 135]
	Via the BATF2/JAK2/STAT3 signaling pathway	Facilitate relieve ankylosing spondylitis	[136]
miR-26a	Related to the TLR4/NF-κB signaling pathway	Inhibit the proliferation of high glucose-induced human skin fibroblasts cells, and promote cell apoptosis	[137]

*MSCs* mesenchymal stem cells, *TRIM14* tripartite motif containing 14, *PI3K* phosphatidylinositol 3-kinase, *TRAIL* TNF-related apoptosis inducing ligand, *MDA* MDA-MB-231 cells, *Fas-L* Fas ligand, *MM* multiple myeloma, *EMT* Epithelial-mesenchymal transition, *MMP7* matrilysin; matrix metalloproteinase 7, *TGF-β* transforming growth factor-beta, *BATF2* basic leucine zipper transcriptional factor ATF like 2, *JAK* Janus kinase, *STAT* signal transducer and activator of transcription, *TLR4* Toll-like receptor 4.

### MSCs promote apoptosis of immune cells and other cells

MSCs also alleviate various autoimmune diseases by promoting apoptosis of immune cells. The pro-apoptotic effect of MSCs on immune cells is usually regarded as part of the immunosuppressive ability of MSCs. And emerging evidences suggest that MSCs significantly inhibit T cells to exert immunosuppressive activity, which is involved in a variety of immune molecules, including indoleamine (2,3)-dioxygenase, programmed cell death 1 ligand 1, and Fas-L [18, 131–133]. These immune molecules help MSCs effectively alleviate graft-versus-host disease, systemic sclerosis, and DSS-induced ulcerative colitis. In addition, in vitro experiments also find that MSCs promote hepatic stellate cells apoptosis and help to alleviate the process of liver fibrosis [134, 135]. Many ncRNAs are also involved in the remission of autoimmune diseases. MSCs-derived exosomes suppress miR-5189-3p to facilitate the apoptosis of fibroblast-like synoviocytes via the basic leucine zipper ATF-like transcription factor 2/JAK2/STAT3 signaling pathway, which facilitate relieve ankylosing spondylitis (AS) [136]. The MSCs-secreted miR-26a inhibit the proliferation of high glucose-induced human skin fibroblasts cells and promote cell apoptosis, which may be related to the TLR4/NF-κB signaling pathway [137]. These above evidences indicate that the pro-apoptotic effect of MSCs on immune cells is also an integral part of the efficacy of MSCs.

## Self-regulation of apoptosis by MSCs

The apoptosis of MSCs themselves is closely related to the therapeutic effects of MSCT and the treatment of various diseases. Regulation of MSCs apoptosis involves multiple molecular and signaling pathways (Table 3). Several factors, such as hypoxia/serum deprivation, hydrogen peroxide, dexamethasone, and metformin, can induce MSC apoptosis [138–141]. Nonetheless, there are various measures that can alleviate this apoptotic effect, including drug induction, exogenous factor pretreatment, overexpression of genes or ncRNAs, and more [142–145]. Therefore, The primary factors that pose a threat to MSC apoptosis are hypoxia, oxidative stress, and drug toxicity, while the rescue measures primarily target key signaling molecular pathways, including ERK, MAPK, NRF2, PI3K/AKT, and the Bcl-2 family.

**Table 3:** Molecules Inducing Apoptosis in MSCs and Corresponding Rescue Methods.

Pro-apoptotic molecules	Rescue methods	Mechanisms	Refs
Hypoxia/Serum deprivation	Overexpression of Lnc Tmem235	Regulate the miR-34a-3p/BIRC5 axis	[138]
	Andrographolide	Via NRF2 signaling pathway	[142]
	Overexpression of miR-21, miR-23a and miR-210		[165]
	Adjunction of adenosine triphosphate	prevent caspases 3/7 activation and modulate ERK1/2 and p38 MAPK signaling pathways	[166]
	BNIP3-mediated mitophagy	Promote fatty acid synthase -induced free fatty acid synthesis	[167]
	Overexpression of LncAABR07053481	Regulate the miR-664-2-5p/Notch1 pathway	[168]
H_2_O_2_	IL-11	Activate STAT3 signaling	[139]
	PGE1 pretreatment	Regulate the HIF pathway	[143]
	Overexpression of NMNAT3	Improve mitochondrial function and enhance antioxidative stress capacity by NMNAT3-NAD + -SIRT3 axis	[144]
	SDF-1 pretreatment	Activate the pro-survival AKT and ERK signaling pathways and upregulate Bcl-2/Bax ratio	[169]
	Overexpression of miR-210	Through antioxidation and c-Met pathway activation	[170]
	Exendin-4 pretreatment	Through the PI3K/AKT–secreted frizzled-related protein 2 pathway	[171]
	Melatonin/ Astaxanthin	Via NRF2 signaling pathway	[172, 173]
	Fucoxanthin	Through the PI3K/AKT/ NRF2 pathway	[174]
Dexamethasone	Baicalin	Active the hedgehog signaling pathway	[140]
	Eicosapentaenoic acid	Via GPR120-meditated induction of adaptive autophagy	[175]
Metformin		Facilitate osteogenesis and inhibit adipogenesis	[141]
LNC_000052	Upregulation of miR-96-5p	Via the PI3K/AKT signaling pathway	[145]
Glutamate	Repress prostate apoptosis response-4 gene expression		[176]
TNF-α	Silence RUNX2	Inhibit caspase-3 activity and Bax expression	[177]
CoCl_2_ and SIN-1	Transfection of miR-302d	Inhibit CCL-5 expression	[178]
Diabetic serum	Blockade of the C5a/C5aR pathway	Down-regulation of FADD and the Bax/ Bcl-2 ratio	[179]
High glucose stress	AURKA	Transcriptional regulation for FOXO3a	[180]
Ropivacaine	Sufentanil	Regulate miR- 182-5p/BCL10/CYCS	[181]
Ionizing radiation	Redd1 overexpression	Attenuate mitochondrial ROS generation and promote cell autophagy	[182]
Methylprednisolone	Parkinson disease protein 7	Via NRF2 signaling pathway	[183]

*MSCs* mesenchymal stem cells, *miR* micro RNA, *BIRC5* baculoviral IAP repeat-containing protein 5, *NRF2* Nuclear factor-erythroid 2 related factor 2, *ERK* extracellular regulated protein kinases, *MAPK* mitogen-activated protein kinase, *BNIP3* Bcl-2/adenovirus E1B 19 kDa protein-interacting protein 3, *H*_*2*_*O*_*2*_ hydrogen peroxide, *PGE1* prostaglandin E2, *HIF* hypoxia-inducible factor, *NMNAT3* nicotinamide mononucleotide adenylyl transferase 3, *SDF-1* Stromal cell-derived factor 1, *NAD*^*+*^ nicotinamide adenine dinucleotide, *SIRT3* Sirtuin 3, *PI3K* Phosphatidylinositol 3-kinase, *GPR120* G protein coupled receptor 120, *TNF-α* tumor necrosis factor-alpha, *RUNX2* Runt-related transcription factor 2, *CoCl*_*2*_ cobalt chloride, *SIN-1* 3-morpholinosydnonimine hydrochloride, *CCL-5* C-C motif chemokine ligand 5, *C5a/C5aR* complement C5a/C5a receptor, *FADD* Fas-associated protein with death domain, *Fas-L* Fas ligand, *BCL10* B-cell lymphoma/leukemia10, *CYCS* cytochrome c, somatic.

Although MSCs undergo apoptosis due to various reasons, interestingly, apoptotic MSCs also possess significant biological functions and therapeutic potential. Researches have shown that the MSCs injected into the body during MSCT therapy undergo widespread apoptosis and induce receptor-mediated immune regulation within the body, which is closely related to the therapeutic effects of MSCT [146, 147]. Further researches highlight that apoptotic vesicles derived from MSCs (MSC-ApoVs) possess various functions and promising applications, including immune regulation, promotion of proliferation and tissue regeneration, homeostasis maintenance, and drug delivery[148, 149]. This is mainly based on the molecules transferred by MSC-ApoVs [147, 150], and the immune response after immune cell engulfment of MSC-ApoVs [151, 152]. These findings broaden the therapeutic strategies of MSCT and deepen our understanding of MSC-ApoVs.

Moreover, MSCs have demonstrated the ability to exert biological effects through the engulfment of apoptotic cells. This phenomenon occurs when apoptotic cells stimulate MSCs to target apoptotic sites via the HGF/c-Met axis [153, 154]. Furthermore, the presence of circulating apoptotic bodies contributes to the self-renewal and osteogenic differentiation of bone marrow MSCs by delivering cytokines [155]. Additionally, apoptotic cells induce MSCs to actively suppress T-cell immunity through the COX2/PGE2 axis [156]. Consequently, exploring the interaction between MSCs and apoptosis will provide valuable insights into the biological functions of MSCs and the therapeutic potential of MSCT.

## Insights into the dual regulatory effects of MSCs on apoptosis

MSCs exhibit a dualistic characteristic in the regulation of apoptosis, displaying both inhibitory and promotive effects that can be attributed to various factors. Initially, depending on the specific cytokine stimulation, MSCs can exert diverse or even contradictory effects. For instance, interferon-gamma activates MSCs and promotes anti-inflammatory and antiapoptotic effects by inducing the release of various anti-inflammatory factors and growth factors that inhibit inflammatory responses and reduce cell death [157]. Conversely, certain cytokines such as LPS may stimulate MSCs to secrete signaling molecules that enhance inflammation and increase apoptosis [158]. In such cases, MSCs may contribute to promoting inflammatory responses and facilitating apoptotic processes.

Subsequently, MSCs are characterized by their heterogeneity and encompass various subsets that bestow them with remarkable adaptability. Sun et al. and Wang et al. identified 7 tissue-specific and 5 functionally conserved subsets of MSCs using scRNA-seq, demonstrating that hUC-MSCs possess enhanced immunomodulatory potential [159, 160]. Additionally, Zhang S et al. discovered two distinct subsets of MSCs in hUC-MSCs with variations in immune regulation and tissue differentiation functions [161]. The presence of these diverse subsets of stem cells contributes to the bidirectional regulation of the immune system [2] and may also account for the dual regulatory effect on apoptosis.

## Conclusion

The great potential of MSCs in the treatment of various diseases is attracting more and more attention, which makes the research on the therapeutic mechanism of MSCs more and more in-depth. The regulation of apoptosis is an important part of the MSCs therapeutic mechanism. In general, MSCs have been shown to inhibit apoptosis and promote survival of various tissue cells. Moreover, this process involves three major apoptotic regulatory pathways and is closely related to autophagy, aging, and proliferation. However, in partial disease states, MSCs will also show the promotion of apoptosis in specific cells, such as lymphocytes and tumor cells. And these pro-apoptotic effects are generally considered to be part of the immunosuppressive effect of MSCs. These complex and orderly regulatory mechanisms constitute the homeostasis regulatory network of MSCs on tissue cells, which is the basis for MSCs to exert various therapeutic effects. However, the mechanism of MSCs regulating apoptosis still needs to be further explored. In particular, the complex regulatory network of MSCs on apoptosis, autophagy, aging, proliferation and survival of tissue cells deserves more attention. These studies will help to further understand the important role of MSCs in maintaining homeostasis.
